# NLRP3 (rs10754558) gene polymorphism and tumor necrosis factor alpha as predictors for disease activity and response to methotrexate and adalimumab in psoriasis

**DOI:** 10.1186/s12865-024-00630-2

**Published:** 2024-07-04

**Authors:** Fatma Z. Kamel, Heba Allah Mohamed Hoseiny, Aya A. El Shahawy, Ghada Boghdadi, Alia A. El Shahawy

**Affiliations:** 1 Department of Medical Microbiology and Immunology, Faculty of Medicine, Zagazig University, Zagazig, Egypt; 2 Department of Dermatology and Venerology, Faculty of Medicine, Zagazig University, Zagazig, Egypt; 3 Department of Clinical Pathology, Faculty of Medicine, Zagazig University, Zagazig, Egypt; 4 College of Medicine, Ibn Sina National College, Jeddah, Saudi Arabia

**Keywords:** NLRP3, Psoriasis, Biological agents, TNF

## Abstract

**Background:**

Psoriasis has a global prevalence of 1–3%, with variations observed across different ethnic groups and geographical areas. Disease susceptibility and response to anti-tumor necrosis factor-α (TNFα) drugs suggest different genetic regulatory mechanisms which may include NLR family pyrin domain containing 3 (NLRP3) polymorphism. Evaluation of the NLRP3 gene polymorphism, the serum level of CRP and TNFα in psoriasis patients and assessment of the NLRP3 (rs10754558) gene polymorphism, CRP and TNFα with disease severity and their role as biomarkers for response to Methotrexate and Adalimumab in psoriasis. The study had a total of 75 patients diagnosed with psoriasis vulgaris, who were compared to a control group of 75 healthy individuals.

**Results:**

There was a highly significant difference in NLRP3 genotypes and alleles distribution between psoriasis patients and controls (*P* = 0.002,0.004). The heterozygote genotype GC (OR = 3.67,95%CI:1.75–7.68, *P* = 0.0006), was linked with increased risk of psoriasis. Additionally, The GC genotype was significantly associated with nonresponse to psoriasis therapy (OR = 11.7,95%CI:3.24–42.28, *P* = 0.0002). Regarding serum CRP and TNFα levels, there was a highly statistically significant difference between psoriasis patients and controls (*P* < 0.0001), and there was also a highly statistically significant difference between responders and non-responders in psoriasis patients regarding PASI 50 (*P* < 0.0001).

**Conclusions:**

The NLRP3 (rs10754558) genotypes GC was associated with the severe form of psoriasis and with nonresponse to psoriasis medication. Therefore, NLRP3 (rs10754558) gene polymorphism is an important prognostic biomarker in psoriasis patients. The serum TNFα can be used as a predictor for response to therapy in psoriasis patients. More research for evaluation of role of the NLRP3 gene polymorphism in the genetic risks and treatment outcomes associated with psoriasis is still required.

## Introduction

Psoriasis is an immune-mediated disease that affects the whole body and is characterized by inflammation in the skin and joints [1]. Psoriasis is a condition characterized by excessive production of the outermost layer of the skin, resulting in the formation of localized red, itchy, and scaly patches [2]. In more severe cases, these patches can extend to cover the entire body. Psoriasis has a global prevalence of 1–3%, with variations observed across different ethnic groups and geographical areas. Psoriasis is a complex disease with multiple causes, including genetic factors (specifically related to the immune system or skin) and non-genetic factors. The non-genetic factors include imbalances among CD4 + T cell subsets, which contribute to the thickening and abnormal shedding of skin cells, as well as environmental risk factors such as exposure to UV radiation, certain medications, smoking, alcohol consumption, infections, and stress [3].

There is a synchronized connection between the innate and adaptive immune system. The impact of the NLR family pyrin domain containing 3 (NLRP3) inflammasome on this connection has been demonstrated in many studies [4]. The NLRP3 inflammasome is a complex protein structure that starts a type of cell death associated with inflammation and stimulates the release of proinflammatory cytokines IL-1β and IL-18 [5]. The NLRP3 inflammasome has been associated with several diseases, such as psoriasis [3]. The NLRP3 (rs10754558) gene polymorphism serves as a significant diagnostic biomarker in individuals with psoriasis and has the potential to be a valuable tool in therapeutic therapy [2].

Psoriasis can be treated using several methods, including topical medications, phototherapy, conventional systemic medicines like methotrexate, small molecules, and biologics. Various therapies exhibit different levels of effectiveness, safety, speed of response, and long-term response sustainability. They also differ in terms of treatment administration processes, including frequency, duration, and convenience, as well as treatment costs [6]. Referral to a dermatologist may be necessary for the examination and the use of systemic therapy in cases that are more severe and resistant [7].

Various genetic regulatory systems, including NLRP3 polymorphism, could potentially be involved in disease susceptibility and response to anti-tumor necrosis factor-α (TNFα) medications [4]. The association of NLRP3 (rs10754558) gene polymorphism, TNFα and CRP with disease severity and their role as biomarkers for response to different systemic therapy in psoriasis have not been explored to date. To investigate this a critical issue, the aim of this research is to evaluate the NLRP3 (rs10754558) gene polymorphism, the serum level of CRP and TNFα in psoriasis patients and assessment of the NLRP3 (rs10754558) gene polymorphism, CRP and TNFα with disease severity and their role as biomarkers for response to Methotrexate and Adalimumab in psoriasis.

## Methodology

### Study design and subjects

This case-control study with a sample size of 75 individuals diagnosed with psoriasis vulgaris and 75 healthy individuals serving as a control group, that was calculated at confidence interval (CI) 95% and power 80% [8]. Inclusion criteria involved patients aged 18–60 years old, clinically diagnosed with psoriasis, and exhibiting varying degrees of psoriasis vulgaris severity. The medical professional conducted a comprehensive assessment, including a detailed medical history, a thorough examination of the body and skin, and an analysis of the location and spread of the skin lesions, both on the central body and the limbs. The disease severity and extent were clinically assessed using the psoriasis area and severity index (PASI) score. The PASI score is derived from a subjective assessment of the skin lesions, taking into account the severity of symptoms such as redness, swelling, and flaking, as well as the proportion of the affected skin area. Mild psoriasis is characterized by a PASI score of less than 10, while moderate psoriasis falls within the range of 10 to 20. A PASI score higher than 20 indicates severe psoriasis [9, 10].

Seventy-five psoriatic patients presented for the first time with moderate to severe psoriasis. According to the records, some of the selected patients received intramuscular methotrexate by starting dose of 7.5 mg weekly and titrated to a maximum dose of 0.4 mg/kg/week according to clinical response for maximum 6 months while the others received adalimumab at 80 mg as a starting dose on day 1 then 40 mg after 1 week then 40 mg every other week. The median duration of treatment in patients received methotrexate was 9 months (range:3–48 months), whereas the median duration of treatment in patients received adalimumab was 3 months (range:2–4 months). In those patients’ sheets, baseline PASI score was calculated and recorded before starting the therapy while therapeutic PASI score was assessed 3 months after the end of the treatment course. Responder to therapy is defined from calculation PASI 50 response which means achievement of at least 50% reduction in PASI score from baseline, but treatment failure is defined when an improvement of PASI of 50% is not achieved [11].

This study was conducted at the Immunology Research Laboratory, Faculty of Medicine, Zagazig University. Cases were recruited from patient records of the psoriasis unit at the dermatology department of Zagazig University Hospitals, Faculty of Medicine, Zagazig, Egypt. The study took place between 2022 and 2023. The study excluded any subject that had one or more of the following conditions: chronic inflammatory diseases such as tuberculosis, aspergillosis, and chronic hepatitis; systemic immunological disorders such as systemic lupus erythematosus, rheumatoid arthritis, and systemic sclerosis; malignancy; ischemic heart diseases; heart failure; and cerebrovascular events.

### Ethical approvals

This research has been approved by the Institutional Review Board (IRB). Prior to their enrollment in this study, all individuals provided written consent. This research was conducted in accordance with the Helsinki Declaration. The approval number for this study is (IRB#11,123- 17/10/2023).

### NLRP3 (rs10754558) gene polymorphism analysis

Following the manufacturer’s instructions, genomic DNA was extracted from 2 milliliters of EDTA-anticoagulated venous blood using a genomic DNA extraction kit (GeneJET, Thermo Scientific, USA, Cat. no. K078). The concentration and purity of the extracted genomic DNA were measured using the Nanodrop 2000 (Thermofischer Scientific, Wilmingiton, DE, USA) at 260 nm absorbance with a ratio between (1.7–1.8) for A260 /A280. The samples were stored at -20oC until they were needed.

Real-Time PCR was used to assess the polymorphism of the NLRP3 (rs10754558) gene (Applied Biosystems®7500, USA). To prepare the genotyping reaction mix, TaqMan universal master mix II (2x) was used. Table 1 displays the NLRP3 rs10754558 primer sequence. Utilizing TaqMan® MGB probes with a reporter dye at each probe’s 5´ ends, VIC® dye was coupled to the Allele 1 probe’s 5´ end, and 6FAMTM dye was linked to the Allele 2 probe’s 5´ end.

**Table 1:** NLRP3 (rs10754558) primers

Polymorphism	PCR sets
NLRP3(rs10754558) primers	Forward (5’- CCTGAGCTGACCGTCGTCTT-3’) Reverse (5’ATGAGGTCACCAAGAGGAACATC-3’)

### Serum TNFα levels

In the immunology research lab, serum samples were drawn 3 months after the end of the treatment under strict aseptic conditions and stored at -20 °C until analysis. ELISA [Bioassay Technology Laboratory, China] was used to detect TNFα in accordance with the manufacturer’s instructions. Every sample was processed in duplicate, and an ELISA reader (Stat Fax ® 303 Plus) was used to calculate the mean absorbance. To determine the concentration of “serum TNF-α,” calibration curves were employed. The measurements of serum TNFα were made in units of ng/L, with a sensitivity limit of 1.52 ng/L.

### Serum CRP levels

The nephelometric method (Quest Diagnostics, Madison, NJ, USA), with an analytical sensitivity of 0.2 mg/L, was used to measure the serum concentrations of CRP.

### Statistical analysis

The quantitative variables were tested for normality. Comparisons between quantitative variables were performed using independent sample t-tests for parametric data or Mann–Whitney test and Kruskal Wallis test for nonparametric data. The genotype/allele frequencies were analyzed by chi-square (χ2) test. Odds rations (ORs) and 95% CIs were determined whenever χ2 or Fisher’s exact test was significant Correlation between levels of TNF-α in serum and PASI score in psoriasis patients were assessed using Spearman’s correlation coefficient. Statistical significance was defined as having P values below 0.05. The results were obtained using the Statistical Package of Social Services (SPSS, version 22, Chicago, IL, USA). The MedCalc Software Ltd. Odds Ratio (OR) Calculator is used to determine the OR and CI 95% (https://www.medcalc.org/calc/odds_ratio.php (Version 22.018; accessed January 17, 2024).

## Results

### Baselines characteristics of study groups

Our study involved 75 psoriasis patients, A total of 37 females (49.3%) and 38 males (50.7%), the mean age in all patients was 36.33 ± 9.16 years and 75 controls with 41females (54.7%) and 34 males (45.3%) and the mean age in all controls was 35.83 ± 9.62 with no significant difference between the studied groups regarding gender and age (*P* = 0.624,742).

The baselines characteristics of psoriasis patients were shown in Table 2. Although there was no statistically significant difference between responders and non-responders as regards age, gender, family history, duration of treatment, baseline PASI score and therapeutic PASI (*P* > 0.05), there was a statistically significant difference as regards severity (*P* = 0.002). In the responders, there were 18(60%) moderate cases and 12(40%) severe cases while there were 4 (8.9%) mild cases,10(22.2%) moderate cases,31(68.9%) severe cases in the non-responders.

**Table 2:** Baseline characteristics of psoriasis cases (*N* = 75)

	Cases (*n* = 75)	Responder$\dag$ 30(40%)	Non responder 45(60%)	*P* value
Age(yrs.), mean ± SD	36.33 ± 9.16	38.20 ± 9.20	35.09 ± 9.02	0.151
⁰Gender, n (%)				
Male Female	38(50.7%) 37(49.3%)	15(50%) 15(50%)	23(51.1%) 22(48.9%)	1.000
⁰Family history, n (%)				
Yes No	45(60**%**) 30(40**%**)	17(56.7%) 13(43.3%)	28(62.2%) 17(37.8%)	0.810
⁑Duration of disease (yrs.), median (IQR)	4(6)	3.5(4.25)	4(6.5)	0.301
•Severity, n (%)				
Mild Moderate Severe	4(5.3%) 28(37.3%) 43(57.3%)	0 (0%) 18(60%) 12(40%)	4 (8.9%) 10(22.2%) 31(68.9%)	0.002*
•Psoriatic arthritis, n (%)				
Yes No	12(16**%**) 63(84**%**)	2(6.7%) 28(93.3%)	10(22.2%) 35(77.8%)	0.108
⁑Baseline PASI score, median (IQR)	28(24)	22.5(26)	30(25)	0.854
⁑Therapeutic PASI score, median (IQR)	30(32)	15(26)	32(28)	0.121
•Treatment, *n* (%)				
Methotrexate Adalimumab	69(92%) 6(8%)	28(93.3%) 2(6.7%)	41(91.9%) 4(8.9%)	1.000
⁑Treatment duration(mons), median (IQR)	9(18)	8(6)	12(18)	0.053

$\dag$PASI 50 score; n, number; SD, Standard Deviation

⁰Chi-square

⁑Mann-Whitney test

•Fisher’s Exact test; PASI, Psoriasis Area and Severity Index

### NLRP3 genotypes and alleles among the studied groups

Genotypic frequencies of NLRP3 (rs10754558) gene were noticed in line with Hardy-Weinberg equilibrium in controls (χ^2^ = 2.56; *P* = 0.11) and cases (χ^2^ = 2.39; *P* = 0.12). There was a highly significant difference in NLRP3 genotypes and alleles distribution between psoriasis patients and controls (*P* = 0.002,0.004). The most predominant genotype in psoriasis patients was GC (58.7%) compared to GG (52%) in controls as shown in Table 3. The percentage of allele G was 69.3%, and 53.3% in controls and psoriasis patients respectively (*P* = 0.005). The carriers of allele C were at risk of psoriasis by 1.98 times (OR = 1.98, 95%CI:1.22–3.17, *P* = 0.005). Furthermore, the GC genotype (OR = 3.67, 95%CI:1.75–7.68, *P* = 0.0006), was linked with increased risk of psoriasis.

**Table 3:** Distribution of NLRP3 (rs10754558) genotypes and alleles among the studied groups

NLRP3	Cases (*n* = 75)	Controls (*n* = 75)	*P*-value	OR (95% CI)
Genotype⁰ *n*, (%)				
GG	18(24%)	39(52%)	Reference	
CC	13(17.3%)	10(13.3%)	0.042*	2.82(1.04–7.62)
GC	44(58.7%)	26(34.7%)	0.0006*	3.67(1.75–7.68)
GC + CC	57(76%)	36(48%)	0.0005*	3.43(1.71–6.89)
Allele⁰ *n*, (%)				
G	80(53.3%)	104(69.3%)	Reference	
C	70(46.7%)	46(30.7%)	0.005*	1.98(1.22–3.17)

OR, Odds Ratio, CI, Confidence Interval

⁰Chi-square (*p* = 0.002,0.004)

*P < 0.05 is significant

Relations between NLRP3 (rs10754558) gene with several clinical characteristics in psoriasis cases were shown in Table 4. Regarding disease severity in psoriasis patients, there was statistically significant difference among NLRP3 genotypes (*P* < 0.001). About 72.7% of GC genotypes were severe while 55.6% of GG genotypes were severe. The distribution of NLRP3 allele and genotypes according to PASI 50 response and methotrexate response were displayed in Tables 5 and 6. The heterozygote genotype was significantly linked with nonresponse to psoriasis therapy (OR = 11.7, 95%CI:3.24–42.28, *P* = 0.0002) and particularly with nonresponse to methotrexate (OR = 86.3, 95%CI:13.02–572.60, *P* < 0.0001).

**Table 4:** Relations between NLRP3 (rs10754558) genotypes with different clinical parameters in psoriasis patients (*n* = 75)

Variables	NLRP3 (rs10754558) genotype			Test of sig	*P* value
	GG (*n* = 18)	CC (*n* = 13)	GC (*n* = 44)		
⁰Gender, n (%)					
Male Female	9(50%) 9(50%)	5(38.5%) 8(61.5%)	24(54.5%) 20(45.5%)	1.043	0.594
⁰Family History, *n* (%)					
Yes No	9(50%) 9(50%)	1(7.7%) 12(92.3%)	8(18.2%) 36(81.8%)	2.316	0.330
⁑ Duration of disease(yrs.), median (IQR)	4(5.5)	3(4.5)	4(6.8)	0.293	0.864
*Psoriatic Arthritis, *n* (%)					
Yes No	3(16.7%) 15(83.3%)	1(7.7%) 12(92.3%)	8(18.2%) 36(81.8%)	0.679	0.826
*Treatment line, *n* (%)					
Methotrexate Adalimumab	16(88.9%) 2(11.1%)	13(100%) 0(0%)	40(90.9%) 4(9.1%)	1.164	0.711
⁑ Treatment duration (mons), median (IQR)	6.5(5)	8(8)	12(25)	13.926	0.001
⁑ Baseline PASI, median (IQR)	36.5(30)	20(8)	30(24)	7.84	0.02
⁑ Therapeutic PASI, median (IQR)	30(32)	12(5)	32(25)	12.325	0.002
⁰Severity, *n* (%)					
Mild Moderate Severe	0(0%) 8(44.4%) 10(55.6%)	0(0%) 12(92.3%) 1(7.7%)	4(9.1%) 8(18.2%) 32(72.7%)	25.32	< 0.001*

n, number

⁰Chi-square

⁑Mann-Whitney Test

PASI, psoriAsis Area And Severity Index

*Fisher’s Exact Test

**Table 5:** Distribution of NLRP3 (rs10754558) genotypes and alleles according to PASI 50 response

NLRP3	Responder$\dag$ (*n* = 30)	Non responder (*n* = 45)	*P*-value	OR (95% CI)
Genotype⁰ n, (%)				
GG	13(43.3%)	5(11.1%)	Reference	
CC	9(30%)	4(8.9%)	0.856	1.16 (0.24–5.53)
GC	8(26.7%)	36(80%)	0.0002*	11.7(3.24- 42.28)
GC + CC	17(56.7%)	40(88.9%)	0.0026*	6.12(1.89- 19.85)
Allele⁰ n, (%)				
G	34(56.7%)	46(51.1%)	Reference	
C	26(43.3%)	44(48.9%)	0.504	1.25(0.65–2.41)

$$\dag$$PASI 50 score

n, number; OR, Odds Ratio, CI, Confidence Interval

⁰Chi-square (*P* = 0.00002,0.504)

*P < 0.05 is significant

**Table 6:** Distribution of NLRP3 (rs10754558) genotypes and alleles according to methotrexate response

NLRP3	Responder$\dag$ (*n* = 28)	Non responder (*n* = 41)	*P*-value	OR (95% CI)
Genotype⁰ *n*, (%)				
GG	14(50%)	2(4.9%)	Reference	
CC	11(39.3%)	2(4.9%)	0.823	1.27(0.15–10.53)
GC	3(10.7%)	37(90.2%)	< 0.0001*	86.3(13.02–572.60)
GC + CC	14(50%)	39(95.1%)	0.0003*	19.5(3.93–96.83)
Allele⁰ n, (%)				
G	31(55.4%)	41(48.2%)	Reference	
C	25(44.6%)	41(48.2%)	0.536	1.24(0.63–2.45)

$$\dag$$PASI 50 score

n, number; OR, Odds Ratio, CI, Confidence Interval

⁰Chi-square (p = < 0.00001,0.536)

*P < 0.05 is significant

**Fig. 1:**
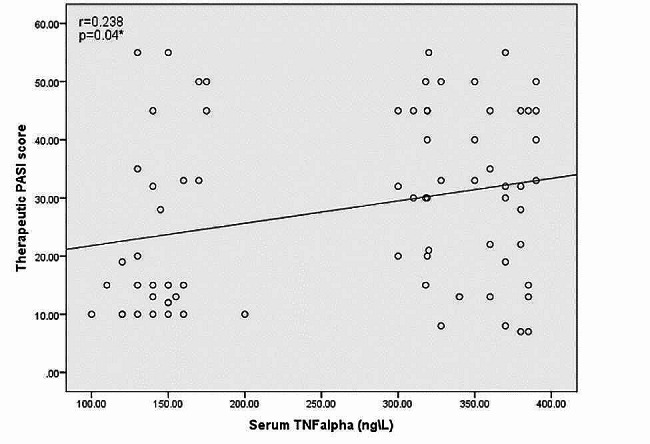
Correlation between levels of TNF-α in serum and PASI score in psoriasis patients. * Correlation is significant at the 0.05 level (2-tailed)

There was highly statistically significant variance between psoriasis patients and controls as regards serum CRP and TNFα levels (*P* < 0.00001). Moreover, there was highly statistically significant variance in serum CRP and TNFα levels between responders and non-responders in psoriasis patients as regards PASI 50 (*P* < 0.0001). While there was no significant difference as regards treatment line (*P* > 0.05) as shown in Table 7. Levels of serum CRP and TNFα in NLRP3 genotypes of psoriasis patients were significantly different (*P* < 0.00001). There was significant correlation between levels of TNFα in serum and PASI score (*p* < 0.05) as shown in Fig. 1.

**Table 7:** Serum levels of CRP and TNF-α among the studied groups

Parameters		CRP		TNF-α	
		Median (IQR)	*P* value	Median (IQR)	*P* value
Groups	Controls⁑ Cases	1.9(1.8) 40.7(40.1)	< 0.00001*	8(8) 318(210)	< 0.00001*
Cases	Treatment line⁑				
	Methotrexate Adalimumab	40.7(40.1) 42.2(40.1)	0.907	318(210) 334.5(218.8)	0.411
	PASI 50 score⁑				
	Responder Non responder	15.4(2.9) 50(21.5)	< 0.00001*	147.5(30) 350(61)	< 0.00001*
	NLRP3 genotype•				
	GG CC GC	15.4(3.07) 15.5(3.4) 50.1(21.5)	< 0.0001*	150(25) 140(35) 350(61)	< 0.0001*

IQR; Interquartile Range

*P < 0.05 is significant

⁑Mann-Whitney U

• Kruskal Wallis test

## Discussion

Psoriasis is an inflammatory illness caused by an immune response. Its exact cause is unknown; however, it may be related to problems with the growth and specialization of skin cells called keratinocytes. This results in infiltration with inflammatory cells, specifically T-lymphocytes, macrophages, and neutrophils. Psoriasis has a prevalence of 1–3% among adults with numerous extracutaneous manifestations [12]. Psoriasis is a persistent and recurring condition that often requires prolonged treatment. The selection of treatment for psoriasis is based on the severity of the disease, the presence of other medical conditions, and the availability of healthcare services. Psoriatic patients are commonly classified into two categories: mild or moderate to severe psoriasis, based on the clinical severity of the lesions, the extent of affected body surface area, and the impact on the patient’s quality of life [13].

The NLRP3 inflammasome is activated in inflammatory skin conditions. Stimulation of NLRP3 inflammasomes has been demonstrated to result in an inflammatory response mostly caused by increased production of IL1b and IL-18. These cytokines play crucial roles in both host defense and inflammation by activating innate immune responses [3]. This study aimed to evaluate the NLRP3 (rs10754558) gene polymorphism, the serum level of CRP and TNFα in psoriasis patients and assessment of the NLRP3 (rs10754558) gene polymorphism, CRP and TNFα with disease severity and their role as biomarkers for response to Methotrexate and Adalimumab in psoriasis. Non statistically significant difference concerning age and gender was noticed between the studied groups (*P* > 0.05). This was consistent with Parisi et al. who reported that psoriasis affects any age, but most cases occur before 35 years old [14]. Furthermore, Guillet et al. found that the prevalence and manifestation of psoriasis of the skin are similar between different sexes [15].

This research revealed a significant statistical association between genotypes GC, CC, and allele C, and an increased susceptibility to psoriasis. The prevalence of Genotype GC and CC in psoriatic patients was 58.7% and 17.3%, respectively, compared to 34.7% and 13.3% in controls. This demonstrates that the presence of Genotype GC raises the risk of psoriasis by about 3.7 times, while the presence of Genotype CC elevates the risk by about 2.8 times. Moreover, the presence of allele C was associated with a 1.9-fold increase in the risk of psoriasis. These results were consistent with an Egyptian study that conducted by **ALrefai**et al. [3]. The finding of another interesting Egyptian study displayed that the GC genotype was the predominant genotype in individuals with psoriasis, accounting for 60.9% of patients compared to 34.4% in the control group. This genotype was associated with a six-fold increase in the susceptibility to psoriasis. The prevalence of genotype CC in psoriatic patients was 23.4%, whereas it was 9.4% in controls. This genotype was associated with a nearly 9-fold increase in the susceptibility to psoriasis. Furthermore, allele C exhibited a significant prevalence in individuals with psoriasis (53.9%) compared to the control group (26.6%), hence increasing the susceptibility to psoriasis by approximately three times [2]. Although our result was similar to **Farag**et al. in prevalence in Genotype GC in psoriasis patients compared to controls, there was difference in other findings. This can be explained by difference of inclusion criteria of study population. Our study only included moderate to severe psoriasis. The prevalence of GG genotype (24%) was higher than prevalence of CC genotype (17.3%) in psoriasis patients in our study while in **Farag**et al. prevalence of CC genotype (23.4%) was higher than prevalence of GG genotype (15.6%) in psoriasis patients. Additionally, severe cases of psoriasis patients in this study represented about 72.7% of GC genotype and 7.7% of CC genotype while in **Farag**et al. represented 10.3% of GC genotype and 40% CC genotype.

The gene polymorphism of NLRP3 (rs10754558) has the potential to influence both the stability and expression of its mRNA. The presence of the rs10754558 C allele leads to a decrease in the stability of NLRP3 mRNA, as compared to the rs10754558 G allele [16]. The NLRP3 rs10754558-G allele is associated with elevated IL-18 levels, indicating that this single nucleotide polymorphism (SNP) may regulate the activation of inflammasomes and contribute to undesirable inflammatory consequences [17]. There was a statistically significant difference in disease severity among psoriasis patients based on their NLRP3 genotypes (*P* < 0.001). This research found a strong association between the GC genotype of NLRP3 and severe psoriasis. Conversely, ALrefai et al. and Farag et al. observed a strong association between the GC genotype of NLRP3 and mild psoriasis. This can be attributed to the difference in number of patients distributed according to severity [2, 3].

An experimental study in a mouse model of imiquimod-induced psoriasis concluded that treatment with an inflammasome blocker caused reduced expression of pNF-kB, pSTAT-3, IL-6, and TNFα [18]. These results reveal that NLRP3 inflammasomes play an important role in psoriatic inflammation and therapy. Current evidence-based guidelines from Europe and national authorities recommend the use of topical medications for treating mild cases of psoriasis. According to Girolomoni et al. traditional synthetic (TS) medicines are the preferred initial therapy for moderate to severe disease. Biologic therapies are advised only if TS drugs are not well-tolerated or do not effectively control the disease [19]. According to Jiang et al. there is an opinion that administering biologics at an early stage of the disease could potentially counteract the inflammatory effects related to psoriasis comorbidities, hence impacting long-term outcomes [20].

Consequently, it is fundamental to predict the response of psoriasis patients to systemic therapy. This study is the first to investigate the association between the NLRP3 (rs10754558) gene polymorphism and response to psoriasis therapy. The study revealed a significant association between the GC genotype of NLRP3 (rs10754558) with nonresponse to psoriasis medication. The OR was 11.7, with a 95% confidence interval (CI) of 3.24–42.28, and a p-value of 0.0002. This was consistent with Awni et al. who studied the role of NLRP3 rs4612666 gene polymorphism as a predictor for anti-TNF α-responsiveness in rheumatoid arthritis patients. They reported that NLRP3 rs4612666 gene polymorphism was related with higher disease activity and lower response to TNFα -inhibitors [4].

A highly statistically significant difference between psoriasis patients and controls was noted as regards serum CRP and TNFα levels (*P* < 0.00001). Moreover, there was highly statistically significant difference in serum CRP and TNFα levels between responders and non-responders in psoriasis patients as regards PASI 50 (*P* < 0.0001). Similarly, Farshchian et al. reported that baseline CRP levels among psoriatic patients were higher than normal [21]. Moreover, Strober et al. and Demir et al. concluded that clinical response in psoriasis patients was associated with greater CRP reductions [22, 23]. Concerning TNFα levels in response to psoriasis therapy, several studies found that serum levels of TNFα in patients with psoriasis were significantly higher than in the control group. Besides, Furiati et al., 2019 reported that patients on methotrexate or anti-TNF therapy produced significantly lower levels of TNFα [4, 24–28].

The results of this study showed a significant (*p* < 0.05) association between the PASI score and serum TNF-α levels in psoriasis patients. Our result agreed with Abdel-Hamid et al.and Hagag et al. who reported that the difference in severity level (PASI) was found to be significantly correlated with serum TNFα level, supporting the role of this cytokine as main parameter for disease severity [27, 29]. On the other hand, Ovcina-Kurtovic and Kasumagic-Halilovic revealed that in psoriasis patients, there was no statistically significant association (*p* > 0,05) between the serum level of TNFα and the PASI score. This may be due to the small sample size of patients and controls, different populations, and genetic factors [28]. The GC genotype of NLRP3 showed the highest serum CRP and TNFα levels (50.1 mg/L, 350 ng/L) which was in line with highest therapeutic PASI score. Additionally, about 72.7% of severe cases were GC genotype. This means that this GC genotype of NLRP3 linked with disease severity and the serum levels of CRP and TNFα can be used as marker for disease activity.

Limitations of the study: the numbers of some subgroups are too small based on disease severity and therapy, which may reduce our analytical power. We recommend further research in more wide scale. Another limitation is that other lines of biological treatment were not included in the study and patient adherence to prescribed regimens between registry visits was not evaluated.

## Conclusion

The NLRP3 (rs10754558) genotypes GC was associated with the severe form of psoriasis and with nonresponse to psoriasis medication. Therefore, NLRP3 (rs10754558) gene polymorphism is an important prognostic biomarker in psoriasis patients. The serum TNFα can be used as a predictor for response to therapy in psoriasis patients. More research for evaluation of role of the NLRP3 gene polymorphism in the genetic risks and treatment outcomes associated with psoriasis is still required.

## Data Availability

The datasets used and/or analysed during the current study are available from the corresponding author on reasonable request.
